# CCR5 D32 modifies 15-year mortality risk associated with well-established clinical and immunological factors among HIV-infected patients

**DOI:** 10.1186/1758-2652-13-S4-P151

**Published:** 2010-11-08

**Authors:** M Parczewski, M Leszczyszyn-Pynka, D Bander, A Urbañska, M Kaczmarczyk, S Ciechanowicz, A Boron-Kaczmarska

**Affiliations:** 1 Department of Infectious Diseases and Hepatology, Pomeranian Medical University, Arkoñska 4, Szczecin, Poland; 2 Pomeranian Medical University, Department of Molecular Medicine, Szczecin, Poland

## Purpose of the study

In the era of long term follow up of HIV+ patients analysis of factors modifying risk of death is vital. The aim of the study was to evaluate predictive value of selected parameters in a cross-sectional analysis.

## Methods

Epidemiological (age, gender, date of HIV diagnosis, route of transmission), clinical (date and reason of AIDS, date and reason of death, baseline viral load, history of cART) and immunological (baseline, nadir, zenith lymphocyte CD4 counts, time of CD4 count > 500 cells/ml) data from 506 patients followed-up from 1996 to 2010 in the Department of Infectious Diseases and Hepatology, Szczecin, Poland were collected with CCR5 D32 genotyping performed for every subject. 15-year survival analysis was performed using Kaplan-Meier methodology with log rank test and univariate Cox regression for hazard ratio calculation. Selected data were implemented into a Cox multivariate model and p< 0.05 assumed of statistical significance.

## Results

Cumulative 15-year mortality rate was 18,5% (95% CI 0,15-0,21), with 64 (68,8%) of AIDS associated and 30 (32,2%) non-AIDS deaths. At 15 year timepoint factors associated with higher probability of survival were CCR5 D32/wt genotype (78,3% vs 65,3%, p = ,033, univariate [univ.] HR=2,21, p=,044), female gender (81,7% vs 62,5%, p=,014; univ. HR=1,98, p=,015), AIDS-free at HIV diagnosis (70,6%vs 59,3%, p <0,01; univ. HR=2,8, p<0,01) and no AIDS during observation (79,4% vs 50,8%, p<0,01, univ. HR=3,58, p<0,01), introduction of cART (74,4% vs 34,2%, p <0,01; univ. HR=3,68, p<0,01), baseline lymphocyte CD4 count >50 cells/ml (71,3% vs 56,5%, p <0,01, univ. HR=2,52, p<0,01), lymphocyte CD4 nadir >50 cells/ml (75,8% vs 55,9%, p = ,00007, univ. HR=2,31, p<0,01), CD4 zenith >500 cells/ml (85,1 % vs 52,8%, p <0,01, , univ. HR=5,22, p<0,01), stable lymphocyte CD4 count > 500 cells/ml for one year (88,2% vs 60,4%, p<0,01, univ. HR=7,59, p<0,01). In multivariate model with gender (HR=1,86 , p=,04), cART initiation (HR=4,62 p<0,01), lymphocyte CD4 zenith >500 (HR=3,86, p<0,01), nadir > 50 (p=n.s) and history of AIDS (HR=2,2 p=,03) positive effect of CCR5 D32/wt on survival was confirmed (HR=2,2, p=,046).

## Conclusions

CCR5 D32 mutation proves to improve survival of HIV+ patients acting in concert with clinical and immunological factors, however data suggest that interventions related to early testing and treatment would strongly influence survival in HIV+ patients.

